# Nematicidal, Acaricidal and Plant Growth-Promoting Activity of *Enterobacter* Endophytic Strains and Identification of Genes Associated with These Biological Activities in the Genomes

**DOI:** 10.3390/plants11223136

**Published:** 2022-11-16

**Authors:** Bernardo Sachman-Ruíz, Arnoldo Wong-Villarreal, Liliana Aguilar-Marcelino, Luis Fernando Lozano-Aguirre, Saúl Espinosa-Zaragoza, Ana Laura Reyes-Reyes, Diana Sanzón-Gómez, Ana Isabel Mireles-Arriaga, Rodrigo Romero-Tirado, Marisol Karina Rocha-Martínez, Juan Diego Pérez-de la Rosa, Ricardo Sánchez-Cruz, Jaime Adriel Gómez-Gutiérrez

**Affiliations:** 1 CENID-Salud Animal e Inocuidad, Instituto Nacional de Investigaciones Forestales Agrícolas y Pecuarias, Jiutepec 62550, Mexico; 2 División Agroalimentaria, Universidad Tecnológica de la Selva, Carretera Ocosingo-Altamirano, km 0.5, Ocosingo 29950, Mexico; 3 National Center for Disciplinary Research in Animal Health and Safety (INIFAP), Km 11 Federal Road Cuernavaca-Cuautla, Jiutepec 62550, Mexico; 4 Centro de Ciencias Genómicas, Universidad Nacional Autónoma de México, AP565-A, Cuernavaca 62210, Mexico; 5 Facultad de Ciencias Agrícolas, Universidad Autónoma de Chiapas, Huehuetán 30660, Mexico; 6 National Institute of Forestry Agricultural and Livestock Research (INIFAP), Campo Experimental Rosario Izapa, Tuxtla Chico 30870, Mexico; 7 Departamento de Agronomía, División Ciencias de la Vida, Campus Irapuato-Salamanca, Universidad de Guanajuato, Irapuato 36500, Mexico; 8 Servicio Nacional de Sanidad, Inocuidad y Calidad Agroalimentaria (SENASICA), Carretera Federal Cuernavaca-Cuautla No. 8534, Colonia Progreso, Jiutepec 62550, Mexico; 9 Centro de Investigación en Biotecnología, Universidad Autónoma del Estado de Morelos, Cuernavaca 62209, Mexico; 10 Centro de Investigaciones Biológicas, Universidad Autónoma del Estado de Morelos, Cuernavaca 62209, Mexico

**Keywords:** phosphate solubilization, auxin production, plant growth promotion, biocontrol, siderophore

## Abstract

In the present study, the nematicidal and acaricidal activity of three *Enterobacter* endophytic strains isolated from *Mimosa pudica* nodules was evaluated. The percentages of mortality of *Enterobacter* NOD4 against *Panagrellus redivivus* was 81.2%, and against *Nacobbus aberrans* 70.1%, *Enterobacter* NOD8 72.4% and 62.5%, and *Enterobacter* NOD10 64.8% and 58.7%, respectively. While against the *Tyrophagus putrescentiae* mite, the mortality percentages were 68.2% due to *Enterobacter* NOD4, 64.3% due to *Enterobacter* NOD8 and 77.8% due to *Enterobacter* NOD10. On the other hand, the ability of the three *Enterobacter* strains to produce indole acetic acid and phosphate solubilization, characteristics related to plant growth-promoting bacteria, was detected. Bioinformatic analysis of the genomes showed the presence of genes related to IAA production, phosphate solubilization, and nitrogen fixation. Phylogenetic analyzes of the *rec*A gene, phylogenomics, and average nucleotide identity (ANI) allowed us to identify the strain *Enterobacter* NOD8 related to *E. mori* and *Enterobacter* NOD10 as *E. asburiae*, while *Enterobacter* NOD4 was identified as a possible new species of this species. The plant growth-promoting, acaricidal and nematicidal activity of the three *Enterobacter* strains makes them a potential agent to include in biocontrol alternatives and as growth-promoting bacteria in crops of agricultural interest.

## 1. Introduction

Agricultural pests, due to phytopathogenic nematodes and dust mites, cause economic losses in crops of great importance [1]. Some species of phytopathogenic nematodes such as *Pratylenchus* spp., *Ditylenchus* spp., *Psilenchus* spp., and the false root-knot nematode *Nacobbus aberrans*, together with *Meloidogyne* spp., (the latter made up of 98 species, including *M. chitwood*, *M. incognita*, *M. hapla*, *M. javanica* and *M. arenaria*), are responsible for causing economic losses of between 12 to 20% in the crops where they occur [2]. Another organism that causes problems in agricultural production, causing considerable damage and losses in food products of plant and animal origin, is the dust mite *T. putrescentiae*, since they can carry fungal spores and pathogenic bacteria for humans. The *T. putrescentiae* mite is a vector of pathogenic bacteria of *Klebsiella* spp., *Candida albicans* and *Staphylococcus* which affect humans, and are present in the mucosa and parts of the skin [3].

Plants are responsible for selecting their microbiome to have beneficial bacterial colonizers designated as plant growth-promoting bacteria (PGPB). These produce different compounds such as auxins, antibiotics, and organic acids that are related to growth promotion, as well as chitinase, protease and cellulase enzymes that are related to biocontrol, health and plant development processes [4,5,6,7,8]. The ability of endophytic bacteria to colonize the interior of plant tissues gives them an advantage in avoiding the competition present in the rhizosphere environment, and allows them to achieve a close relationship with the plant [6,9,10]. The *Enterobacter* genus comprises species that have been reported to promote plant growth due to their multiple growth-promoting activities. Some strains that have been reported are: *E. asburiae* PDA 134 isolated from date palms [11], *E. cloacae* isolated from citrus and corn plants [12,13] and *E. asburiae* from sweet potato [14]. The strain *Enterobacter* sp., P23 has also been reported as a growth promoter under conditions of abiotic stress such as salinity, due to its high ACC deaminase activity, extreme pH, high temperature and in the presence of a wide variety of pesticides traditionally applied in rice, peanut and corn crops [15,16].

On the other hand, different species of the genus *Enterobacter* have been evaluated against phytoparasitic nematodes of agricultural pests. In this context, in a study by Zhao et al. [17], the nematicidal activity of *E. ludwigii* AA4 was reported in 98.3 and 98.6% against the pine nematode *Bursaphelenchus xylophilus*, considering *E. ludwigii* AA4 as a biocontrol agent of the pine nematode (*Pinus sylvestris*) infection. Regarding root-knot nematodes, a study by Oh et al. [18] reported the in vitro nematicidal activity of *E. asburiae* against eggs and J2 larvae of *M. incognita*. The results were obtained at a concentration of 10% of the treatment against the eggs, and 53.7% and 98.2% against J2 larvae in a time of two to seven days post-treatment.

Considering the characteristics of the species of the genus *Enterobacter*, the objective of this work is to analyze the genome sequences of three strains isolated from *Mimosa pudica* nodules to identify the genes that are associated with the characteristics that promote plant growth, nematicidal activity and acaricide, as well as its phylogenetic relationship with other species of the genus *Enterobacter*.

## 2. Results

### 2.1. Genome Assembly and Annotation

The analysis of the genomes by means of the average nucleotide identity (ANI) was obtained by comparing the sequences of the genomes against the genomes deposited in GenBank. The NOD4 strain was identified as *Enterobacter* sp. with 88.14% with a coverage of 82%, NOD8 as *E. mori* with 96.46% with a coverage of 87.72%, and NOD10 as *E. asburiae* with 97.12% with a coverage of 87.80%. The strain *Enterobacter* sp. NOD4 contains 242 contigs with a genome size of 4.65 Mpb, containing 53.1% GC and 4378 coding sequences, while the *E. mori* NOD8 strain contains 244 contigs with a genome size of 4.84 Mpb and 55.7% GC content and 4403 coding sequences. Finally, the *E. asburiae* NOD10 strain contains 144 contigs with a genome size of 4.51 Mpb and 56.1% GC content and 4198 coding sequences (Table 1).

### 2.2. Phylogenomic Analysis

**Figure 1:**
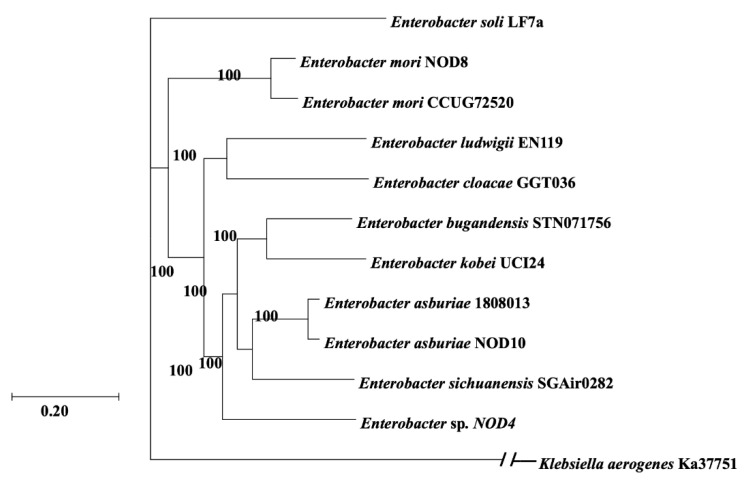
Molecular Phylogenetic Analysis of Enterobacter with *rec*A gene by Maximum Likelihood Method based on the General Time Reversible Model. The tree with the highest log likelihood (−3614.36) is shown. Initial tree(s) for the heuristic search were obtained automatically by applying Neighbor-Join and BioNJ algorithms to a matrix of pairwise distances estimated using the Maximum Composite Likelihood (MCL) approach, and then selecting the topology with superior log likelihood value. A discrete Gamma distribution was used to model evolutionary rate differences among sites (5 categories (+G, parameter = 0.1689)). The rate variation model allowed for some sites to be evolutionarily invariable ([+I], 39.76% sites). The tree is drawn to scale, with branch lengths measured in the number of substitutions per site. The analysis involved 18 nucleotide sequences. All positions containing gaps and missing data were eliminated. There was a total of 879 positions in the final dataset. Evolutionary analyses were conducted in MEGA X.

The sequences of the *rec*A genes were analyzed using the BLASTn algorithm and showed similarity with the species of the *Enterobacte*r genus. Phylogenetic trees were constructed using the *rec*A genes. Phylogenomic analysis shows that the NOD4 strain may be a new species of this genus. The NOD8 strain is phylogenetically related to the *E. mori* species, while the NOD10 strain is related to the *E. asburiae* species (Figure 1). This is also observed with the BLAST results, where a 99% similarity with the aforementioned species was obtained.

### 2.3. Phosphate Solubilization Activity

**Figure 2:**
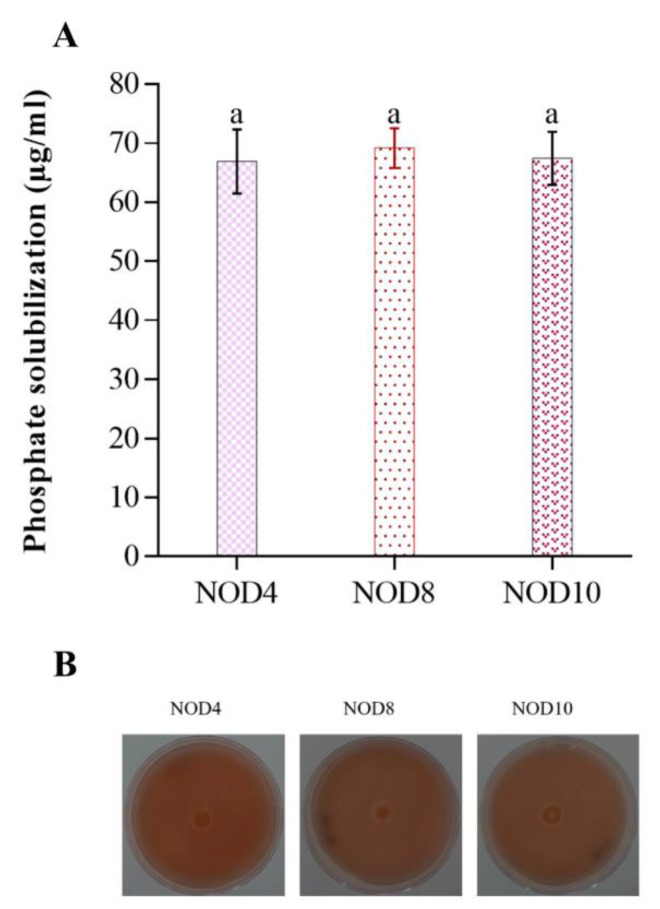
Solubilization of inorganic phosphate by the three strains of *Enterobacter*. (**A**) Phosphate solubilization (**B**) Pikovskaya PVK.

The three strains of *Enterobacter* had the ability to solubilize phosphate, *Enterobacter* sp. NOD4 solubilizes 66.9 μg/mL, while *E. mori* NOD8, 69.2 μg/mL, and *E. asburiae* NOD10, 67.4 μg/mL at five days. The statistical analysis does not show a significant difference (ANOVA *p* < 0.05) in phosphate solubilization between strains (Figure 2A). Phosphate solubilization can also be observed through the formation of halos in Pikovskaya PVK agar culture medium (Figure 2B).

### 2.4. Determination of Auxin Production

**Figure 3:**
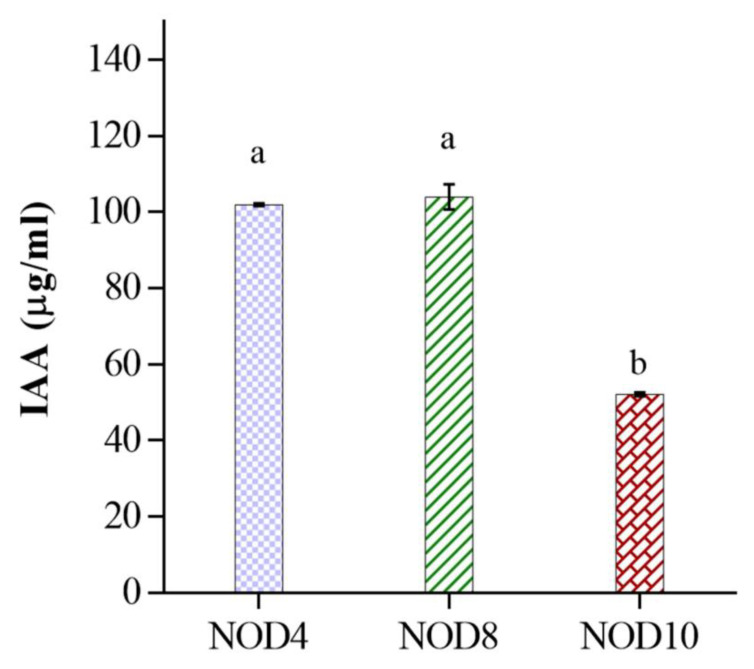
IAA quantification of the three *Enterobacter* strains. Media was supplemented with tryptophan. Different symbols at the top of the bars indicate a statistically significant difference (*p* < 0.05).

The production of indoles by bacteria is a mechanism for promoting plant growth. This characteristic was detected in *Enterobacter* sp. NOD 4 producing 101.9 μg/mL, *E. mori* NOD8, 103.9 μg/mL and *E. asburiae* NOD10 52.1 μg/mL (Figure 3). In the statistical analysis of the indole production by the strains, a significant difference was found (ANOVA *p* < 0.05) in the NOD 4 and NOD 8 strains, with respect to the NOD 10 strain.

### 2.5. Siderophore Production

**Figure 4:**
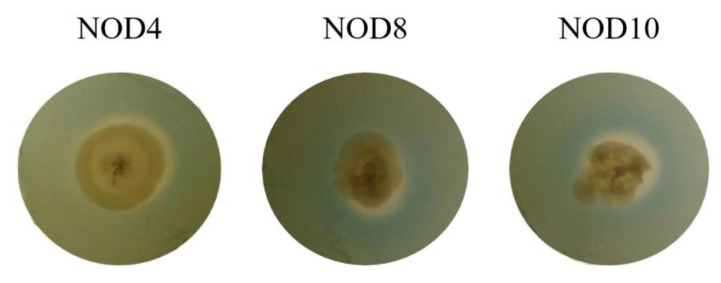
Determination of the production of siderophores in the three strains of *Enterobacter*.

The *Enterobacter* sp. NOD4, *E. mori* NOD8 and *E. asburiae* NOD10 were inoculated in the CAS-CAA medium, where they formed a yellow halo around the colonies indicating the production of siderophores. The three strains were positive (Figure 4).

### 2.6. In Vitro Evaluation of Enterobacter Strains against P. redivivus and N. aberrans

The results of the evaluation of the strains of *Enterobacter* sp. NOD4 at a concentration of 1 × 10^9^ cell/mL against the free-living nematode *P. redivivus* showed a mortality of 81.2%, *E. mori* (NOD8) of 72.4% and *E. asburiae* (NOD10) of 64.8% at 24 h (Table 2). The strain of *Enterobacter* sp. NOD4 against the nematode *N. aberrans* showed a mortality of 70.1%, *E. mori* (NOD8) of 62.5% and *E. asburiae* (NOD10) of 58.7% at 24 h (Table 3).

### 2.7. In Vitro Evaluation of Enterobacter against the Mite T. putrescentiae

The results of the in vitro evaluation of *Enterobacter* against the mite *T. putrescentiae* are described below. The strain of *Enterobacter* sp. NOD4 at a concentration of 1 × 10^9^ cell/mL against the *T. putrescentiae* mite showed a mortality of 68.2%, the *E. mori* strain (NOD8) at the same concentration of 1 × 10^9^ cell/mL showed a mortality of 64.3%. *E. asburiae* (NOD10) at the same concentration of 1 × 10^9^ cell/mL, 79.8% was obtained, and ivermectin at a concentration of 2.5 mg/mL, 99.3% mortality at 24 h (Table 4).

### 2.8. Bioinformatic Analysis of Genomes for the Detection of Genes Involved in Plant Growth-Promoting Activities and Biocontrol

In the genome of the *E. asburiae* NOD10 strain, the *asp*C gene was identified, encoding an enzyme that catalyzes the conversion of indole-3-pyruvic acid to indole-3-acetaldehyde. This is later converted into IAA by the enzyme aldehyde dehydrogenase (*ald*B), that was also detected in the genome of *E. asburiae* NOD10 (Table 5). Other genes that were detected in *Enterobacter* sp. NOD4, *E. mori* NOD 8 and *E. asburiae* NOD10 are *nir*D which codes for the minor subunit of the enzyme nitrite reductase, *nar*I which codes for the alpha subunit of the enzyme nitrate reductase, *nas*R which regulates the assimilation of nitrates and nitrites, and *nar*X that detects the presence of nitrites and nitrates, the *amt*B gene that codes for an ammonium transporter, and the *nif*J gene that codes for pyruvate-flavodoxin-oxidoreductase. All of these genes are involved in nitrogen fixation processes (Table 5).

Thus, the *gln*K gene is involved in the regulation of nitrogen metabolism. In the bioinformatic analysis of the *Enterobacter* strains, the *pst*S gene that codes for the phosphate-binding protein and the *pho*U gene that is involved in the regulation of phosphate assimilation were also detected (Table 5). The *ent*H gene was also identified in the bioinformatic analysis as a corrector in the synthesis of the enterobactin siderophore (Table 5).

## 3. Discussion

In the present study, biotechnological characteristics of interest were detected such as the production of indoles and phosphate solubilization, as well as the production of siderophores related to biocontrol activity. Nematicidal (*P. redivivis* and *N. aberrans*) and acaricidal (*T. putrecentiae*) activity was also detected in the three *Enterobacter* strains. In the bioinformatic analysis of the genomes sequenced in this work, genes involved in the solubilization processes of phosphate, indoles and siderophores were detected.

Phylogenetic analysis using the *rec*A gene of the three isolates showed that they belong to the genus *Enterobacter*. Consistently, phylogenomic inference suggests that the *Enterobacter* NOD8 strain is related to *E. mori*, while the *Enterobacter* NOD10 strain is related to *E. asburiae*. However, the *Enterobacter* NOD4 strain is grouped externally from the *E. asburiae-sichuanensis* and *E. bundangensis-kobei* complexes. This could be an indication that the NOD4 isolate is a new species of *Enterobacter*. These results were confirmed with the analysis of the genomes using average nucleotide identity (ANI), which is a similar statistic of the genomes that allows us to identify them at the species level [19,20]. Where the cutoff to consider 96% as a species is established by the ANI analysis, two strains meet this criteria *Enterobacter* NOD8 and *Enterobacter* NOD10 to be considered as *Enterobacter mori* and *Enterobacter asburiae* species, respectively, while the *Enterobacter* NOD4 strain has an ANI of 84%, so it can be considered a new species [21].

In the *E. asburiae* HK169 strain, nematicidal activity against the root-knot nematode *M. incognita* (eggs and J2) was reported Oh et al. [18]. In this work, the nematicidal activity of the strains *E. asburiae* NOD10, *E. mori* NOD8 and *Enterobacter* sp. NOD4 against the free-living nematode *P. redivivus* and the plant parasite *N. aberrans* (J2). The study carried out by Zhao et al. [17] reported that the *E. ludwigii* AA4 strain has activity against the pine nematode *B. xylophilus* and identified the *sda*B gene that codes for L-serine hydrolase that is involved in nematicidal activity.

The biocontrol of the three strains of *Enterobacter* was not only limited to the nematode *N. aberrans*, it also had activity against the dust mite *T. putrescentiae*, being the first report of the species *E. mori* NOD8, *E. asburiae* NOD10 and *Enterobacter* sp. NOD4 against this nematode. Additionally, the nematode *P. redivivus* was used as a study model to evaluate the nematicidal activity of the species *E. mori* NOD8, *E. asburiae* NOD10, and *Enterobacter* sp. NOD4. These previous results can be considered for future field applications, considering the environmental impact of these organisms.

On the other hand, a study by Hall et al. [22] reported the isolation of *Comamonas* as endosymbionts of the *Psoroptes ovis* mite for biocontrol. Saccà and Lodesani [23] reported *Lactobacillus kunkeei*, *Bacillus thuringiensis*, *Bifidobacterium asteroides* and *Acetobacteraceae* isolated from healthy honey bees and dead varroa mites, which had acaricidal activity against *Varroa destructor*. Finally, the strain *B. amyloliquefaciens* W1 and *Burkholderia rinojensis* were reported to have acaricidal activity against *Tetranychus urticae* [24,25].

Other characteristics of biotechnological interest detected in these *Enterobacter* strains was the ability to solubilize phosphate, where the *pst*S gene related to this process was identified. This phosphate-solubilizing activity has been reported in the *E. ludwigii* GAK2 strain and in the *Enterobacter* sp. 49 [8,26]. Indole Acetic Acid (IAA) production activity was also detected in *E. mori* NOD8, *E. asburiae* NOD10 and *Enterobacte*r sp. NOD4. This activity has been reported in *E. ludwigii* GAK2 and *Enterobacter* sp. 49 [8,26]; the aspC gene was detected by bioinformatic analysis in the genome of the strain E. asburiae NOD10. The ability to produce siderophores could be observed in the three *Enterobacter* strains. This ability related to biocontrol has been reported in the *E. roggenkampii* ED5 strain, and is related to the biocontrol of phytopathogenic fungi [27]. Another study by Solanki et al. [28] reported the production of siderophores by the *Enterobacter* sp. strain, which is related to the biocontrol of *Rhizoctonia solani*. In the genomes of the three *Enterobacter* strains the *ent*H gene was identified, which is involved as a corrector in the synthesis of the siderophore enterobactin. Sánchez et al. [29] reported that *Enterobacter* strains detected the production of indole acetic acid, solubilization of phosphate and siderophores, as well as the detection of *nif*H genes.

Although the nitrogen production tests were not carried out in the different strains, the bioinformatic analysis allowed the detection of genes involved in nitrogen metabolism, such as the *nif*J genes involved in nitrogen fixation, the *nar*X gene in the detection of nitrates and nitrite, the *nir*D gene that encodes the minor subunit of the enzyme nitrite reductase involved in the reduction of nitrates to nitrites, as well as the *amt*B gene related to ammonium transport (Table 5). Guo et al. [27] reported the genes involved in plant growth-promoting characteristics in the *E. roggenkampii* ED5 genome. Ludueña et al. [8] reported the genes involved in the production of siderophores, indole acetic acid and phosphate solubilization in the *Enterobacter* sp. J49.

## 4. Materials and Methods

### 4.1. Isolation of Microorganisms from Mimosa Pudica Nodules

Strains NOD4, NOD8 y NOD10 were isolated from *M. pudica* nodules collected in the Lacandon tropical rain forest in Chiapas, México (16°45′0″ N, 91°30′0″ W). Nodules were surface sterilized using 70% ethanol (10 min) and 2% sodium hypochlorite (20 min). Sterile nodules were crushed, subsequently adding 1 mL of 10 mM MgSO4 and the resulting suspension was streaked on PY agar plates (2% peptone, 1% yeast extract, 2% bacteriological agar), which were incubated at 28 ± 1 °C for five days. Axenic cultures were obtained and conserved in 20% glycerol [29].

### 4.2. DNA Extraction, Library Preparation, and Sequencing

Genomic DNA was extracted from freshly cultivated cells of the strains using the ZR Fungal/Bacterial DNA Kit kitTM (Zymo Research (Tustin, California, USA)), according to the manufacturer’s instructions. The libraries for sequencing were performed with de Nextera XT^®^ (Heslin Rothenberg Farley & Mesiti P.C. (Albany, New York, USA)) protocol following the manufacturer’s recommendations. The samples were fragmented obtaining short chains of DNA ~500 bp. The resulting fragments go through an adapter ligation process (index), and were subsequently amplified following the cycling conditions specified in the NexteraXT protocol. At the end of this process, fragment purification was performed using the Agencourt Ampure XB beads commercial system (Beckman Coulter™ (Brea, California, USA)). Once the libraries were obtained, their quality was verified and analyzed by capillary electrophoresis using the 2100 Bioanalyzer (Agilent Technologies Inc. (Santa Clara, California, USA)). Finally, the samples were analyzed on the Illumina MiSeq high-throughput sequencing platform with paired ends.

The quality analysis of the lectures was performed with FASTQC (bioinformatics.babraham.ac.uk/projects/fastqc/ (accessed on 24 February 2021)) and for cleaning TRIM_GALORE (bioinformatics.babraham.ac.uk/projects/trim_galore/ (accessed on 24 February 2021). Genomes were assembled using Spades version 3.12.0 [30]. Genome sequence annotation was made by PROKKA version 1.12 [31]. This Whole Genome Shotgun project has been deposited at DDBJ/ENA/GenBank under the accession isolate 4 JAKKOK000000000 (PRJNA798777), isolate 8 JAKNRT000000000 (PRJNA798778), and isolate 10 JAKKOL000000000 (PRJNA798780). The version described in this paper is version isolate 4 JAKKOK000000000, isolate 8 JAKNRT000000000, and isolate 10 JAKKOL000000000.

### 4.3. Phylogenetics and Phylogenomics

From the phylogeny carried out with the *rec*A gene, we selected the closest and most representative genomes of the sequenced isolates to obtain a complete nucleotide level database of 8 species of *Enterobacter* and one of *Klebsiella* as an outgroup. We used GET_HOMOLOGUES and GET_PHYLOMARKERS, a pipeline designed to identify high-quality markers to estimate robust genome phylogenies from the orthologous clusters, or the pan-genome matrix (PGM). A ML species tree was estimated from the concatenated set of top-ranking alignments at the DNA or protein levels, using either FastTree or IQ-TREE [32]. 

### 4.4. Phosphate Solubilization

The ability to solubilize phosphate was qualitatively determined by inoculating the strains in PY liquid medium with 0.14 mM CaCl_2_ and incubating at 28 °C for 24 h with shaking at 200 rpm to obtain a pre-inoculum. Bacterial cultures were centrifuged and adjusted to 0.2 OD 600 nm. They were seeded in triplicate in NBRIP culture medium (glucose, 1%; Ca_3_(PO_4_)_2_, 0.5%; (NH_4_)_2_SO_4_, 0.01%; MgSO_4_ 7H_2_O, 0.025%; KCl, 0.02%; MgCl_2_·6H_2_O, 0.5%; Congo red, 2.5 mg/mL agar, 1.8%) and incubated at 28 °C for seven days. After this period, the sizes of the halos around the colonies were measured [33]. Inorganic phosphate concentration was quantitatively measured in liquid culture using NBRIP medium without agar and Congo red. The strains were inoculated at 0.2 OD 600 nm and cultured for ten days at 28 °C at 200 rpm. Samples were taken every 48 h, and after centrifugation of the samples, the concentration of phosphate in the supernatant was measured by the method of Rodríguez and Fraga [34]. The phosphate concentration was expressed as 50 µg/mL.

### 4.5. Determination of Auxin Production

To determine the production of indoleacetic acid (IAA), the strains were grown in liquid NFB medium (composition in g/L: malic acid, 5; K_2_HPO_4_, 0.5; MgSO_4_·7H_2_O, 0.2; NaCl, 0.1; CaCl_2_, 0.02; FeSO_4_, 0.015; Na_2_MoO_4_, 0.0025; MnSO_4_, 0.01; KOH, 4.8; NH_4_Cl, 0.2; yeast extract, 0.3; H_3_BO_4_, 0.01); bacterial cultures were incubated for 18 h at 28 °C at 200 rpm. Then, the cultures were adjusted to 0.2 at OD 600 nm, and 100 μL of culture was inoculated into Jain and Patriquin medium (composition in g/L: succinic acid, 2.5; fructose, 2.5; K_2_HPO_4_, 6; KH_2_PO_4_, 4; NH_4_Cl, 1; MgSO_4_, 0.2; NaCl, 0.1; CaCl_2_, 0.02; FeCl_3_, 0.01; NaMoO_4_, 0.002 and KOH, 2.1) with and without tryptophan (0.1 g/L) and incubated at 28 °C for 24 and 48 h at 200 rpm. 1 mL aliquots of the culture were taken, centrifuged for 5 min at 5000 g and 0.5 mL of the supernatant was mixed with 0.5 mL of Salkowski reagent [35].

### 4.6. Siderophore Production Assays

Siderophore production was determined by the method described by Schwyn and Neilands [36]. CAS-CAA (Chrome azurol (100 mM) and S-casamino acids) agar plates were inoculated with the isolates at 28 °C for 12 days. Orange halos formed around the colonies on blue agar, and were considered indicative of siderophore production. 

### 4.7. Panagrellus Redivivus

A strain of the free-living nematode *P. redivivus* (mixed populations) was used by Dr. Roberto-de-Lara of the Autonomous Metropolitan University (UAM, Xochimilco, Mexico in 2009). The nematodes were grown in plastic containers using commercial oat flakes and water as a substrate. The oat flakes and water were mixed and sterilized in a microwave oven [37]. The nematodes were transferred to the substrate. The containers were covered with an aluminum lid with a mesh window of fine cloth to allow oxygenation. The cultures were maintained at room temperature (25–30 °C). After one week, the population of nematodes increased considerably [38].

### 4.8. Nacobbus Aberrans

The inoculum of *N. aberrans* was obtained from tomato roots (monoxenic culture) (Population of Colegio de Postgraduados, Campus Montecillo, Estado de México, México). Egg extraction was carried out following the methodology described by Vrain [39], and obtaining juveniles (J2) was carried out according to Villar et al. [40].

### 4.9. Tyrophagus Putrescentiae

Populations of the *T. putrescentiae* mite were used, whose breeding stock is found in the National Center for Disciplinary Research in Animal Health and Safety, INIFAP, Jiutepec, Morelos. The specimens were isolated in 2013 in the town of San Juan Tlacotenco, Morelos, Mexico. The population is maintained by feeding them the free-living nematode *P. redivivus* (Goodey) for reproduction. The mites are transferred to plates with sterile agar in order to obtain monocultures of mites, and are kept at room temperature (28 ± 2 °C) under dark conditions [38].

### 4.10. Experimental Design

A completely randomized design was used, which consisted of four repetitions and two replicates for each treatment. The treatments for each bioassay were *Enterobacter* NOD4, NOD8 and NOD10 at a concentration of 1 × 10^9^ cell/mL, as a negative control distilled water was used in the tests against the mite *T. putrescentiae* and the phytoparasite *N. aberrans*. As a positive control, Nematrol plus^®^, a commercial nematicide at a concentration of 6 mg/mL for *N. aberrans* was used. In the case of *P. redivivus* and *T. putrescentiae*, ivermectin, a commercial anthelmintic, was used at a concentration of 5 mg/mL, and distilled water was used as a negative control in the *P. redivivus* bioassays. 

For the in vitro tests with *P. redivivus* and *N. aberrans*, 96-well microtiter plates were used. In each well 50 µL of distilled water with 100 nematodes and 50 µL of *Enterobacter* (NOD4, NOD8 and NOD10) were added. They were subsequently incubated in a humid chamber at 28 °C, and the nematicidal activity was evaluated at 48 h. For the in vitro bioassays of *T. putrescentiae*, 24-well microtiter plates were used. In each well, 10 mites were added with 50 µL of *Enterobacter* (NOD4, NOD8 and NOD10). They were subsequently incubated in a humid chamber at 28 °C, and after 72 h the readings were made with a stereoscope (4× and 10×).

### 4.11. Statistical Analysis

The data obtained were normalized using the arcsine square root transformation and analyzed as a completely randomized design. Means were compared using Tukey’s test (using the R^®^ environment). A value of *p* ≤ 0.05 was considered significant [41].

## 5. Conclusions

With the results obtained in the present study, the strains of *Enterobacter* sp. NOD4, *E. mori* NOD8 and *E. asburiae* NOD10, can be alternative biocontrols against the nematode *N. aberrans* and the mite *T. putrescentiae*. The production of IAA and phosphate solubilization were also detected, which are characteristics that promote plant growth and which can be used in soils composed of insoluble phosphate. Therefore, they are candidates for the biocontrol of nematodes and mites in crops, as well as promoting plant growth in crops of agricultural interest.

## Figures and Tables

**Table 1:** Characteristics of the genomes of *Enterobacter* endophytic strains.

Features	*Enterobacter* sp. NOD4	*Enterobacter* sp. NOD8	*Enterobacter* sp. NOD10
Contigs	242	244	144
Genome size	4,649,192 bp	4,835,007 bp	4,517,298 bp
GC content (%)	53.1	55.7	56.1
Coding gene	4378	4403	4198
tRNA	77	80	80
rRNA	17	19	12
tmRNA	1	1	1
Hypothetical proteins	1064	958	965
Proteins with functional assignments	3314	3445	3233

**Table 2:** In vitro evaluation of Enterobacter strains against *Panagrellus redivivus*.

Treatment	Concentration	%Mortality
*Enterobacter* sp. NOD 4	1 × 10^9^ cell/mL	81.2 ^b^ ± 11.08
*Enterobacter mori* NOD8	1 × 10^9^ cell/mL	72.4 ^c^ ± 10.23
*Enterobacter asburiae* NOD10	1 × 10^9^ cell/mL	64.8 ^b^ ± 27.33
Control (Water)	-	2.1 ^d^ ± 3.35
Ivermectin	2.5 mg/mL	99.3 ^a^ ± 4.15

Means with the same letter are not statistically different in Tukey’s mean comparison test (*p* ≤ 0.05).

**Table 3:** In vitro evaluation of *Enterobacter* strains against *Nacobbus aberrans* (J2).

Treatment	Concentration	% Mortality
*Enterobacter* sp. NOD4	1 × 10^9^ cell/mL	70.1 ^b^ ± 9.2
*Enterobacter mori* NOD8	1 × 10^9^ cell/mL	62.5 ^b^ ± 10.2
*Enterobacter asburiae* NOD10	1 × 10^9^ cell/mL	58.7 ^b^ ± 19.4
Control (Water)	-	2.1 ^d^ ± 3.35
Nematrol	6.0 mg/mL	99.3 ^a^ ± 4.15

Means with the same letter are not statistically different in Tukey’s mean comparison test (*p* ≤ 0.05).

**Table 4:** In vitro evaluation of Enterobacter strains against *Tyrophagus putrescentiae*.

Treatment	Concentration	% Mortality
*Enterobacter* sp. NOD 4	1 × 10^9^ cell/mL	68.2 ^b^ ± 7.31
*Enterobacter mori* NOD 8	1 × 10^9^ cell/mL	64.3 ^b^ ± 10.1
*Enterobacter asburiae* NOD 10	1 × 10^9^ cell/mL	77.8 ^b^ ± 15.2
Control (Water)	-	2.1 ^d^ ± 3.35
Ivermectin	2.5 mg/mL	99.3 ^a^ ± 4.15

Means with the same letter are not statistically different in Tukey’s mean comparison test (*p* ≤ 0.05).

**Table 5:** Genes involved in possible plant growth-promoting and antagonistic activities in *Enterobacter* strains.

Gene	Gene Locus NOD4 JAKKOK000000000.1 *Enterobacter* sp.	Gene Locus NOD8 JAKNRT000000000.1 *Enterobacter mori*	Gene Locus NOD10 JAKKOL000000000.1 *Enterobacter asburiae*	Gene Product
pstS	L2X67_10965 36964.38004	L2X83_10375 11318.12358	L2X78_07895 87736.88776	Phosphate-binding protein
phoU	L2X67_10945 33473.34198	L2X78_07875 84186.84911	L2X83_10395 15184.15909	Negative regulatory protein of pho regulon
aspC			L2X83_17980 1562.2752	Catalyzes the conversion of indole-3-pyruvic acid to indole-3-acetaldehyde
nifJ	L2X67_22275 1..2275	L2X78_22070 111.3635	L2X83_09580 13515.17039	Nitrogen fixation protein
hscA	L2X67_06355 33733.35583	L2X78_13325 33980..35830	L2X83_16170 11435.13285	Fe-S protein assembly chaperone HscA
erpA	L2X67_09295 21597.21941	L2X78_18585 13739..14086	L2X83_05375 44667.45014	Iron–sulfur cluster insertion protein ErpA
glnK	L2X67_18100 6179.6517	L2X78_19110 3818.4156	L2X83_18150 3818.4156	P-II family nitrogen regulator
narX	L2X67_19200 1906.3702	L2X78_20400 1883.3679	L2X83_19625 11800.13596	Nitrate/nitrite two-component system sensor histidine kinase NarX
amtB	L2X67_18095 4854.6143	L2X78_19105 2496.3782	L2X83_18145 7443.8732	Ammonium transporter AmtB
nirD	L2X67_10630 32781.33107	L2X78_00765 171713.172039	L2X83_04385 140648.140974	Nitrite reductase small subunit NirD
nasR	L2X67_19240 15543.16727	L2X78_20440 15561.16745		Nitrate regulatory protein NasR
narI		L2X78_20090 2639.3319		respiratory nitrate reductase subunit gamma
aldB	L2X67_04385 31416.32954		L2X83_21280 5772.7310	Aldehyde dehydrogenase AldB
entH	L2X67_04135 81428.81841	L2X78_06195 71878.72291	L2X83_08015 81225.81638	Proofreading thioesterase EntH

## Data Availability

Not applicable.
